# Data on translatome analysis of *Mycoplasma gallisepticum*

**DOI:** 10.1016/j.dib.2016.08.056

**Published:** 2016-09-07

**Authors:** G.Y. Fisunov, D.V. Evsyutina, V.M. Govorun

**Affiliations:** Federal Research and Clinical Centre of Physical-Chemical Medicine, Malaya Pirogovskaya 1a, Moscow 119992, Russia

**Keywords:** Mycoplasma, Transcriptome, Translatome, Translation, Stress

## Abstract

*Mycoplasma gallisepticum* is a bacterium of class Mollicutes which encompasses wall-less bacteria with significantly reduced genomes. Due to their overall reduction and simplicity mycoplasmas serve as a model of minimal cell and are used for systems biology studies. Here we present raw data on translatome (ribosome-bound mRNA) analysis of *Mycoplasma gallisepticum* under logarithm growth and heat stress. The data supports the publication of “Ribosomal profiling of *Mycoplasma gallisepticum*” (G. Y. Fisunov, D. V Evsyutina, A. A. Arzamasov, I. O. Butenko, V. M. Govorun, 2015) [1].

**Specifications Table**

**Table:** 

Subject area	*Biology*
More specific subject area	*Transcriptomics*
Type of data	*Tables*
How data was acquired	*Data was acquired on SOLiD 5500XL System (Life Technologies).*
Data format	*Raw, processed*
Experimental factors	*Mycoplasma gallisepticum S6 cells were cultured as described previously*[2]*until mid-exponential phase. Heat stress was performed at 46 °C.*
Experimental features	*Ribosome-bound RNA extraction was performed as described in*[1].
	*cDNA libraries were constructed according to Life Technologies protocol for RNA-seq.*
	*After that libraries were depleted of ribosomal RNA cDNA by DSN treatment as described in*[2].
	*Sequencing was performed according to Life Technologies protocol for RNA-seq.*
Data source location	*N/A*
Data accessibility	*Data is within this article and raw data was deposited at NCBI SRA database under project id PRJNA301561,*http://www.ncbi.nlm.nih.gov/bioproject/?term=PRJNA301561

**Value of the data**

• The data provide quantitative analysis of ribosome-bound RNA, which may be used both for determination of the absolute amount and fold-change of ribosome-bound RNA under stress.
• Data contributes to the understanding of genetic information transfer and gene expression regulation on the level of translation.
• Data may be used to study stress response on the level that reflects adaptation strategy to the more extent than purely the level of transcription.
• Data may be used to identify determinants for ribosome preferences for mRNA binding.

## 1 Data

The data represent sequence coverage obtained by sequencing of ribosome-bound RNA of *Mycoplasma gallisepticum* (http://www.ncbi.nlm.nih.gov/bioproject/?term=PRJNA301561). Reads were mapped and the coverage data was analyzed as described previously [2]. CP006916.3 genome assembly was used. Quantitative data is summarized on Table 1.

**Protein ID**: Protein ID according to NCBI.

**Gene ID**: Respective gene ID according to NCBI.

**Gene name**:

**rpkm.Control1-3:** relative coverage of the control samples in three biological replicates.

**rpkm.Heat_stress1-3:** relative coverage of the heat stress samples in three biological replicates.

**qv:** statistical significance of the change.

**log2FC:** fold change between the stress and control samples.

Sequence coverage in bedGraph format is presented in Supplementary material 1. Data for each sample is presented for plus and minus strands separately, which is indicated by pos (plus strand) or neg (minus strand) in the file name.

## 2 Experimental design, materials and methods

### 2.1 Cell culturing

*Mycoplasma gallisepticum S6* cells were cultivated and subjected to stress as described previously [2].

### 2.2 Ribosome-bound RNA isolation

Ribosome-bound RNA was prepared as described earlier [1]. Translation was quenched by addition of chloramphenicol to a final concentration of 100 µg/ml. Cells were harvested by centrifugation and lysed in a buffer containing 0.3% of NP-40. Lysates were frozen at −75°C, thawed and the debris was removed by centrifugation. Then the lysates were applied on a sucrose gradient of 10–50% with a step of 10%. Centrifugation was performed on Optima (Beckman Coulter) centrifuge with MLS 50 bucket rotor (Beckman Coulter) with an average centrifugal force of 200620 g for 1 h at 4 °C. The supernatant was divided into 200 µl fractions. RNA was isolated by Trizol LS as described previously [1].

### 2.3 RNA-seq libraries preparation

RNA-seq libraries were prepared as described previously [2]. Briefly, RNA was sheared by zinc sulfate buffer, ligated to adapters and amplified. After that libraries were depleted of ribosomal RNA cDNA using duplex-specific nuclease as described earlier [2]. Samples were prepared in three biological replicates with one technical replicate per one biological replicate.

### 2.4 Sequencing

Sequencing of ribosome-bound RNA samples was performed on a SOLiD 5500XL System (Life Technologies) with reagents for 50 bp reads according to the manufacturer׳s protocol.
