# Supplementary material for: Data on translatome analysis of Mycoplasma gallisepticum
Source: Data Brief. 2016 Sep 7;9:422–4. doi: 10.1016/j.dib.2016.08.056 (PMC5037205; doi:10.1016/j.dib.2016.08.056)
Supplement: Supplementary file 1 — Supplementary material [file mmc2.doc]

Authors declare no conflicts of interest.
